# Analysis of Single Board Architectures Integrating Sensors Technologies †

**DOI:** 10.3390/s21186303

**Published:** 2021-09-21

**Authors:** José Luis Álvarez, Juan Daniel Mozo, Eladio Durán

**Affiliations:** 1 Department of Information Technologies, University of Huelva, 21007 Huelva, Spain; alvarez@dti.uhu.es; 2 Centro Científico Tecnológico de Huelva (CCTH), University of Huelva, 21007 Huelva, Spain; jdaniel.mozo@diq.uhu.es; 3 Department of Chemical Engineering, Physical Chemistry and Materials Sciences, University of Huelva, 21007 Huelva, Spain; 4 Department of Electronic Engineering, Computer Systems and Automation, University of Huelva, 21007 Huelva, Spain

**Keywords:** single-board computers, microcontroller boards, architectures, integrating sensors technologies, indoor comfort monitoring, IoT applications

## Abstract

Development boards, Single-Board Computers (SBCs) and Single-Board Microcontrollers (SBMs) integrating sensors and communication technologies have become a very popular and interesting solution in the last decade. They are of interest for their simplicity, versatility, adaptability, ease of use and prototyping, which allow them to serve as a starting point for projects and as reference for all kinds of designs. In this sense, there are innumerable applications integrating sensors and communication technologies where they are increasingly used, including robotics, domotics, testing and measurement, Do-It-Yourself (DIY) projects, Internet of Things (IoT) devices in the home or workplace and science, technology, engineering, educational and also academic world for STEAM (Science, Technology, Engineering and Mathematics) skills. The interest in single-board architectures and their applications have caused that all electronics manufacturers currently develop low-cost single board platform solutions. In this paper we realized an analysis of the most important topics related with single-board architectures integrating sensors. We analyze the most popular platforms based on characteristics as: cost, processing capacity, integrated processing technology and open-source license, as well as power consumption (mA@V), reliability (%), programming flexibility, support availability and electronics utilities. For evaluation, an experimental framework has been designed and implemented with six sensors (temperature, humidity, CO_2_/TVOC, pressure, ambient light and CO) and different data storage and monitoring options: locally on a μSD (Micro Secure Digital), on a Cloud Server, on a Web Server or on a Mobile Application.

## 1. Introduction

Single-Board Architectures (SBAs) are similar to a computer in terms of the basic components that make them up on a single board: memory, input/output ports and processor. These basic components are included in a single monolithic chip (System on Chip, SoC) and makes SBAs increasingly develop in compact form and at low-cost, in medium and low power, and with high, low and medium processing capacity, making them very popular for data acquisition systems Integrating Sensor Technologies (ISTs) in numerous applications. SBAs can be classified from different points of view, mainly processor that integrate it, sensors that configure it, communication modules, programming languages, cost, size and open or closed-source.

The first architectures based on a single board were developed, commercialized and began to be used in the 1970s, however it has been in the last 20 years when SBAs have reached their greatest role in terms of use, mainly due to their low cost, consumption, small size and great flexibility, which makes them an alternative in many applications.

These types of platforms integrating sensors, communication networks and data processing are of interest in many engineering applications. Currently there are a large number of sensors that can measure almost all the physical or chemical magnitudes of our environment, which results in a large amount of data to process to define these variables accurately. Therefore, the sensors and the subsequent processing of the data provided are fundamental in Electrical, Electronics, Chemical and Mechanical Engineering, Information Technology, Robotics and Automation, Consumer Electronics as well as emerging applications such as Internet of Things (IoT), Industry 4.0, Intelligent Vehicles and Smart Cities.

The interest in SBAs (and ISTs also) is reflected in the numerous publications and sub-cited analysis, both from the academic, research and economic/business world [1,2,3,4,5,6,7,8,9,10,11,12,13] as well as from industry and manufacturers publications [14,15,16,17,18,19,20]. The most recent forecasts (taking into account pre-COVID and post-COVID scenarios) estimate that the market for SBAs is expected to increase at a Compound Annual Growth Rate (CAGR) of 12.0% in the next five years, from US$632.10 million in 2019 to US$1010.78 million in 2026 [21,22,23,24,25,26,27,28,29,30,31,32,33,34,35,36]. This growth is mainly due to sectors such as Do-It-Yourself (DIY) projects, academic world for STEAM (Science, Technology, Engineering and Mathematics) skills and IoT, where interconnected devices is a reality, including the transition from 4G to 5G technologies. These estimates contemplate various aspects and point of view such as technology, service, processor, application, end use, as well as increased of modules, embedded systems and sensors integrating, the development of the end-use industries such as energy, transportation and medical. This focus on SBAs and ISTs applications has also led to the development and commercialization of low-cost single board platform solutions by all electronics manufacturers and suppliers [37,38,39,40,41,42,43,44,45,46,47,48,49,50,51,52,53,54,55,56,57,58,59,60,61,62,63,64,65,66,67,68,69,70,71,72,73,74,75,76,77,78,79,80,81,82,83,84,85,86].

The applications of Single Board Architectures Integrating Sensors Technologies (SBA-ISTs) cover a wide range in education, industrial and engineering sceneries, from educational tools and entertainment to healthcare, medicine and hospitals, test and measurement, aerospace, military and defense, research, industrial automation, logistics transportation and communication. In engineering, they allow the evaluation of the experimental prototyping and design process. The ability to perform rapid prototyping and exploration of ideas on an easy-to-use development platform is an important factor in software development. In the industry, they allow rapid manufacturing and are part of embedded systems. In test and measurement, they allow the prior validation of the processes. In aerospace, military and defense a variety of designs based on SBAs and ISTs are developed. In the training, education and academic world enables the implementation of project-based learning and for STEAM skills, which enables to build and maintain technology. In healthcare, medicine and hospital facilities, they allow from customized medical monitoring kits and remote monitoring to collects data and processing for tracking of blood pressure sensors, recording lung and heart microphones, oximeters and temperature sensors. In entertainment applications, SBA and IST platforms are a reality and suit a wide and diverse group of potential projects, such as: game consoles, media servers, home security, digital audio hubs, IoT and automation devices, remote desktop and sensor gateways. The growth rate of the electronics industry has also been partially driven by SBAs and ISTs applications, due to their increased demand.

**Figure 1:**
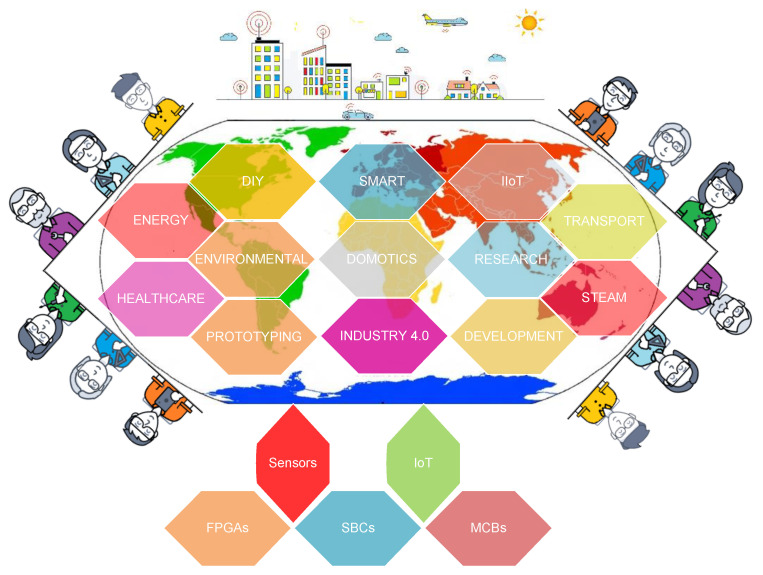
Applications of Single Board Architectures Integrating Sensors Technologies.

The importance of single board architectures integrating sensors technologies and their applications has led to different tutorials, overviews, reviews and surveys papers being reported in the literature, included on-line publications, white papers, webinars and different aspects, topics and points of view: SoC technologies, process capacity, characteristics, power and number of I/O, in terms of cost, control flexibility, among others. In this sense SBAs and ISTs for visual sensor networks are revised and analyzed in [87,88], transport technologies and related applications in [89,90] including tracking, monitoring and lighting in [91,92,93,94,95,96,97,98], SBAs and ISTs for smart cities applications in [99,100], education and research projects in [101,102,103,104,105], medical devices in [106,107,108], smart and advanced sensors in [109,110,111,112,113], including wireless sensors [114], IoT applications in [115,116,117,118], smart home in [119,120,121,122], energy in [123,124,125], engineering education in [126,127,128], Hybrid Electric Vehicle (HEV), Fuel Cell Electric Vehicles (FCEV) and Plug-in-Hybrid Electric Vehicle (PHEV) in [129,130], while reviews and characteristics evaluation are addressed in [131,132]. Figure 1 presents a perspective of different applications where several SBAs and ISTs are utilized.

In this paper, we realized an overview analysis of the most important topics related to SBA-ISTs comparing their potential based on their characteristics.

The work is organized as follows: Section 2 reviews the different single board architectures integrating sensors based on Single-Boards Microcontrollers (SBMs), Single-Boards Computers (SBCs) and Single-Boards with FPGAs (Field Programmable Gate Arrays) and some of their characteristics are analyzed. Section 3 defines an experimental framework and discuss the most important aspects that involves its design, development and implementation based on the obtained results. Low-cost single board architectures integrating low-cost sensors commercially available are used. Finally in Section 4 some conclusions are presented.

## 2. Single Board Architectures Integrating Sensors Technologies (SBA-ISTs)

**Figure 2:**
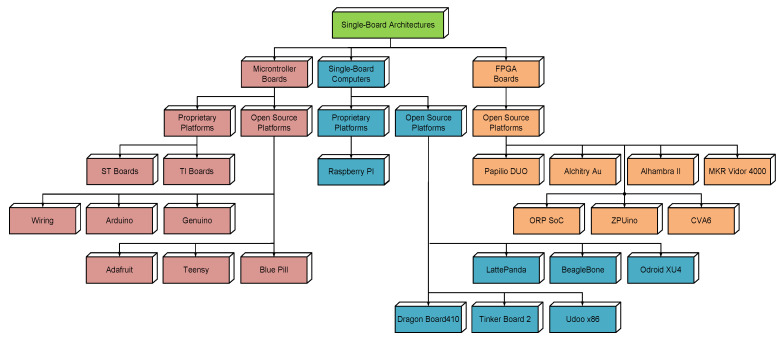
Single Board Architectures classification and main platforms.

A first classification of development systems with single-board architecture (SBAs) can be made according to the type of processor used in their structure. Thus, three types of SBA development systems can be distinguished: SBMs, SBCs and boards based on FPGAs, as shown in Figure 2.

In general terms, SBCs are the most versatile and reliable, usually they support an Operating System (OS) such as Linux or Windows; and have much higher computational capacity than SBMs. In turn, the latter are more focused on electronics and are oriented to the management of inputs and outputs ports. As an example, in IoT devices the equipment connected to sensors for data collection should be governed by microcontrollers, while those for processing the amount of input information will be a microcomputer.

The PCB (Printed Circuit Board), interconnection cables and development software tools together form the development kit. These boards also usually include pins, connectors and expansion sockets to interface the system with other devices.

On the other hand, there are a set of more or less common peripherals that allow expanding the capacity of these systems, although there are very depending on the SBA manufacturer. For each development board/platform there are different expansion boards designed to properly plug into the pins/connectors. Therefore, expansion boards are specific to a particular development kit and, to distinguish them from others, they are often named with specifically names. As an example, Arduino expansion boards are named “shield” and Raspberry Pi ones’ “hat”.

Regardless of the type of SBA considered, the manufacturer will try to cover any type of needs that the user may have and will offer very low-cost systems with limited but sufficient performances, high-capacity systems somewhat more expensive and of course intermediate level systems. This means that the variety of possibilities offered by the market is increasing and, in many cases, requires prior analysis to decide the best option for a specific project. The tables below list some devices and a selection of their most important features in an attempt to facilitate this task.

### 2.1. Hardware Development Platforms: Single-Board Microcontroller (SBMs)

The low-cost of microcontrollers together with the low cost of PCB manufacturing has given rise to a large number of hardware development platforms, both proprietary and open. The success of these platforms is based on two fundamental aspects: the first is the low cost of the hardware; the second is the availability of an Integrated Development Environment (IDE) software with a multitude of libraries and a community of developers that facilitate the resolution of different problems.

The limited capabilities of this type of processors (small memory capacity, 8 or 16-bit data bus) means that they are usually used in stand-alone applications: the microcontroller regulates the operation of a relatively simple device by means of a small program recorded in its memory that is executed continuously (also called “firmware”). Table 1 summarized the most popular proprietary SBMs available in the market and some important characteristics. 

In addition to proprietary SBMs shown in Table 1, it is worth mentioning other manufacturers and SBM families such as the development kits based on Microchip’s 8/16/32-bit PIC microprocessors [63], Intel’s 32-bit Galileo [56] or those marketed by Maxim [61] or Cypress [47] to evaluate their chips. These SBMs are generally oriented more towards evaluating and demonstrating the capabilities of the microprocessors that they integrate, rather than using them as the basis for low-cost technology development. The idea of the manufacturers is that the chips are integrated as such in the developments rather than using the SBM as a part of the development. In the same way Table 2 summarized the most popular open-source SBMs available in the market and some important characteristics.

Among the free source SBMs, Arduino is the most distributed SBM platform all over the world, being converted in almost a standard. There are many third-part Arduino-compatible SBMs having the same pinout, shape, size or characteristics, or including increased performance because anyone can modify or adapt the original design to improve it. In addition, there is a broad developer community that have created an enormous number of libraries and resources for any project can be undertaken. This can say also for the plethora of existing expansion boards, the named shield-boards.

The hardware of Arduino boards is a PCB with an Atmel AVR microcontroller (ATmega8, Atmega168, Atmega328, Atmega1280 depending on model) whose IO ports are pin-accessible and includes a minimum of auxiliary components. The boards can be acquired completely mounted or without components, but they can also be edited because their technical files are freely web accessible. By other hand, the software is an IDE based in Processing that can be downloaded freely from web. It uses Wiring, a programming language based on C, to program the processor whose reference information is continuously debugged and commented by an extensive developer community. The Arduino projects can run without connecting to a computer if an interactive autonomous object is developed. However, Arduino can also be connected to software as Processing, Max/MSP, Pure Data, Java, JavaScript and others to run as an auxiliary object in a big comprehensive project.

Even though the Feather Huzzah ESP8266 from Adafruit has been included in Table 2 as if it were an SBM, it is actually a SoC that integrate an enhanced version of Tensilica’s L106 Diamond series 32-bit RISC (Reduced Instruction Set Computer) processor and a full Wi-Fi front-end (both as client and access point) and TCP/IP stack with DNS support as well. On a 3 × 5 cm PCB there are 9 GPIO, analog input, USB, I^2^C, SPI and FDTI communications. These features, the possibility of programming in the Arduino IDE and its low price is that allows comparing it with the other SBMs.

### 2.2. Hardware Development Platforms: Single-Board Computers (SBCs)

In the last years, the microelectronics technological evolution has made possible to manufacture hardware platforms similar to microcontroller, but with two main differences: they include high memory capacity chips (up to Gigas) both RAM and non-volatile (using Flash technology) and use SoC technology that in addition to high-capacity microprocessors (32 and 64 bits) integrated in a single chip, a very large set of peripheral controllers, such as graphical processors (HD or 4K), interfacing protocols (UART, I^2^C, SPI, GPIO, CSI, etc.), wireless, audio, GPS, nine-axis accelerometer, gyroscope and compass, and much more. These enhanced features allow these hardware platforms to run a complete OS without any problem, so that they work practically as a general-purpose computer. These hardware platforms are referred as SBCs. There are many commercial SBCs, and everyone has a characteristic that makes unique. Even the engineers who regularly work with SBCs may be overwhelmed by their expanding market.

Raspberry PI is, perhaps, the SBC that has had the greatest diffusion, becoming, such as Arduino for SBMs, the benchmark of SBCs (see Table 3). Raspberry PI family is based on ARM/Cortex architecture. The most widely used model, Raspberry Pi 3, is based on a 64-bit SoC ARM Cortex-A53 working at 1.2 GHz, and a GPU Broadcom Video Core 4. It has 1 GB of RAM at 900 MHz, and for storage uses μSD cards. They have mainly two different models of Raspberry PI, named Model A (65 mm × 56 mm) and B (85 mm × 56 mm), in addition there is also the Zero series which is half the A size (65 mm × 30 mm). However, Raspberry PI is neither the only SBC nor the one with the highest performance (see Table 4 for comparison). Since 2012, many SBCs have been developed especially designed to work as embedded systems in a multitude of different applications, many of them are completely open designs and some, such as the Raspberry PI, only partially open. The industries of mobile telephony, IoT or domotics, among others, have greatly favored the development of these platforms, which increasingly have more memory capacity, include dual core, quad core, SoCs, have wireless connectivity and are increasingly compact and inexpensive.

As can be seen in Table 4 and Table 5, most of the open-source SBCs on the market have 32 or 64 bit processors, at least 1 GB of RAM and a certain amount of flash memory to contain the firmware, some kind of wireless connectivity, several options for connecting the most commonly used peripherals such as cameras, audio, keyboards and displays, networking capabilities, GPIO (General Purposed Input Output) pins to control devices such as those that would be managed by an SBM, run Linux type or Windows OS and all this for a price ranging from 50 to 100€ depending on the included features.

The BeagleBone Boards, Blue and Black, are focused to hardware applications. The BeagleBone Black has seven analog inputs up to 200 kS per second and an internal RTC (Real Time Clock), making it a very compact and simple system for continuous acquisition systems. Their main characteristics are: ARM Sitara AM3358 processor (1 GHz), 512 MB RAM, Ethernet, SPI, I^2^C, 69 GPIO, 4 timers, 7 analog inputs and other for more specific uses. The Analog-to-Digital Converter (ADC) or Touchscreen Controller, as is named in the AM335x Technique Reference Manual, is a general purpose 12-bits 8-channel ADC with optional support for resistive touchscreens. Among the 8 analog-channels at processor, only 7 are addressable on the BeagleBone Black expansion port by P9 port. As the analog input range is 0 to 1.8 V, a 1.8 V supply voltage pin is available. The C revision of BeagleBone Black has Linux Debian as OS and natively includes a Pyton interpreter. The ease of use of this language makes it a good choice for managing the main application of an acquisition system.

The Tinker Board is equipped with a 4-core Rockchip RK3288 ARM processor, at 1.8 GHz, and 2 GB of dual-channel LPDDR3 memory. In a size of 85.6 mm × 56 mm × 21 mm includes 1 GB Ethernet connectivity, Mali-T764 GPU with HD/UHD video play support, H.264/H.265 decoding, 192 kHz/24-bits audio, 4 USB 2.0 ports, Bluetooth 4.0 and Wi-Fi 802.11b/g/n. The TinkerOS operating system is a Linux distribution based on the latest Debian 9 kernel version. This OS provides a platform for basic tasks as web browsing, video and music playback. In addition, the LXDE desktop includes a Chromium browser and programming applications.

Udoo x86 Ultra is based in an Intel Pentium x86 processor and its performance is comparable to a low-cost computer. It can run all the software available for a computer, including 3D games, graphical editors, video streaming and more, because a Linux, Android or Windows 10 OS can be loaded. However, it includes an Intel Curie as coprocessor, so the word of Arduino 101 can be also accessed, with gyroscope and six-axis accelerometer integrated. Such dual nature makes this SBC a highly versatile tool suitable for any type of application.

With 8 GB of RAM and a quad-core Intel chip at 1.6 GHz, the Udoo x86 Ultra can run an Office Suite, a web browser or an IDE in the same way that a conventional computer. It can also run resource-intensive games at 720p and 20 or 30 Frames Per Second (FPS). The Udoo x86 Ultra stands out as an SBC suitable for media streaming. With a GPU Intel HD Graphics 405 can play 4K video at 30 Hz in three displays simultaneously, using HDMI and two mini-DisplayPort ports. For storage, it has 32 GB of eMMC (embedded Multi Media Card, a sort of SSD incorporated), but a μSD card can be added as additional storage solution. Its cost is higher comparing with the ARM chip-based SBCs, 250€, and ~10€ should be added if a Wi-Fi antenna is needed, as the board does not have hardware for wireless networking or Bluetooth.

In addition, there is a wide variety of industrial-grade SBCs that are used in automation of processes, production systems and quality control, Industrial IoT or Industry 4.0, they have technology protected by patent and we will not discuss in this work about them.

### 2.3. Hardware Development Platforms Based on Field Programmable Gate Arrays (FPGAs)

A FPGA is a chip including a matrix of logical gates whose inputs and outputs can be interconnected by means of a program. This kind of circuit is widely used in digital control equipment because it is a very compact way of having a large number of logical gates and because, being programmable, it allows very easily configuring the response of the circuit according to the requirements of the system. Moreover, being basically a parallel processing system, its response time is much shorter than that of the best processor. Its main disadvantage is that the programming tools provided by manufacturers are complex, heavy, expensive and exclusive to each of them.

Since their appearance in the 80’s they have greatly increased their capacity and speed and since the 2000’s they have started to use their great capacity to integrate other devices such as clocks, communication controllers and microprocessors that can be programmed in the FPGA matrix as it will be a SoC, this fact makes the development and programming tools more and more complex. Until 2015 all this technology, both hardware and software, was proprietary, but the IceStorm project [133] uses reverse engineering to release the technology of Lattice’s iCE40 family. Since that time, the open-source community has access to use this type of technology to develop projects, and very interesting open-source development boards [134] and programming software have started to emerge that facilitate access to this technology while keeping prices at a reasonable level. It is worth highlighting the iCEstudio IDE among the programming tools and the open FPGA-based SBAs listed in Table 6.

As can be seen in Table 6, there are SBA having only the FPGA chip and the peripherals required for its operation: voltage regulator, memory, communication port for programming, GPIO pins and others. On the other hand, there are manufacturers that, in addition to the FPGA, include a microprocessor to give more flexibility and applicability to the SBA. This option can facilitate FPGA programming by using the processor for support and avoiding the FPGA programming IDE. Others such as Arduino go further and include wireless communication (Wi-Fi and BT/BLE), video input and output ports (MIPI and HMDI, respectively) and other functionalities. There are also manufacturers who choose to maintain form and pinout compatibility with Arduino UNO in order to be able to use the myriad of existing expansion boards (shields).

There are also some FPGA-based SoCs projects, which integrate the FPGA and some peripherals in an only chip acquiring the maximum integration and saving size. Some of them are listed in Table 7.

The ZPUino is not specifically a SoC but is a 32-bits soft processor that it was programmed on a FPGA. It uses a variation of the Arduino IDE for programming and is used by the Papilio Pro and Papilio One FPGA evaluation boards as the inner processor over the Xilinx Spartan 6 LX9 and 3E FPGA chips, respectively [139]. On the other hand, the CVA6 (formerly Ariane project) by PULP Platform [140] is not a FPGA but a SoC with a processor that can emulate the FPGA working, by now only the Xilinx’s Genesys 2.

**Table 7:** SoCs that integrate a FPGA.

	ORP SoC [141]	ZPUino [142]	CVA6 [143]
Processor	OpenRISC 1k	Zylin ZPU	RISC-V
FPGA type	Cyclone 3	Spartan 3E / 6	Genesys 2
Flash (MB)	16	-	-
RAM (MB)	32	32	-
Peripherals	Ethernet, UART, LCD/VGA, SD/MMC, GPIO, Audio, PS2	UART, VGA, MMC, GPIO 128 pin, Audio, SPI, I^2^C	Ethernet, UART, DDR3, SPI
OS	Linux	ZPUino IDE	Linux

## 3. Results and Discussion

In this Section we provide an analysis of the most important aspects that involves the design, development and implementation of projects by means low-cost single board architectures integrating low-cost sensors. Our goal is to highlight some of the main features and possibilities that these architectures present today, as well as their evaluation and comparison by means of a specific example.

Thus, we have defined an experimental framework that allows the integration of a sufficient variety of low-cost sensors with different communication protocols, as well as the monitoring of the measured variables, on different low-cost SBAs. In this sense, we have developed and implemented an indoor comfort monitoring system, which has allowed carried out results during the development and monitoring phases.

### 3.1. Experimental Framework

In order to analyze SBAs with different features, we propose an experimental framework that facilitates the use and comparison of the different SBAs in a common practical environment. In this sense, an indoor comfort monitoring example project has been implemented.

This framework focuses on defining the following issues:

1. Define the environmental variables that will be monitored to ensure environmental quality.
2. Establish the sensors to be used according to different communication modes such as SPI (Serial Peripheral Interface), I^2^C (Inter-Integrated Circuit), 1-Wire protocols, analog and digital signals.
3. Define different communication systems and storage methods for the information provided from the sensors.
4. Consider the integration of the auxiliary modules and electronic components needed to make the system functional for a specific SBA.
5. Define the process of monitoring, evaluation and analysis of results.

The paragraphs below show the details of each of these points in depth.

The environmental quality of a home or workplace is a very important factor for the health of its tenants, parameters such as air quality, temperature or humidity and light levels can be responsible for diseases or poor performance of people. The use of sensors to measure these variables combined with Internet can help improve environmental quality and comfort in homes. The variables that influence comfort are mainly related to temperature, relative humidity, atmospheric pressure and the level of lighting. In regard to air quality, carbon dioxide (CO_2_), total volatile organic compounds (TVOC) and carbon monoxide (CO) are of interest and represent the best way to identify environmental quality.

In this sense, our approach addresses monitoring the following environmental variables:

- Temperature (°C),
- Humidity (%),
- Atmospheric Pressure (hPa),
- Carbon Dioxide (CO_2_) (ppm),
- Carbon Monoxide (CO) (ppm),
- Total Volatile Organic Compounds (TVOC) (ppm),
- Lighting (lux = lumes/m^2^).

Respecting the selection of sensors for the monitoring of the defined environmental parameters, we have tried to use commercial low-cost sensor modules. In order to select a suitable sensor, its measurement range, time response, operating conditions, accuracy, cost, weight and size are some important parameters that must be considered. We have also considered the different connection options and communication protocols with the SBAs. Thus, we have selected modules with digital and analog connection, SPI (Serial Peripheral Interface), I^2^C (Inter-Integrated Circuit) and 1-Wire protocol.

Table 8 and Table 9 show the six sensors selected for experimental framework and a summary of their main features: measured variables, connection type, manufacturer, supply voltage and cost.

One of the selection criteria for sensors is their low-cost, and despite this they have high-level technical characteristics. These sensors have a well-defined response range [144,145,146,147,148,149] and they are suitable to measure parameters of typical atmospheric samples, i.e., habitable indoor ambients with contamination levels within the legally enabled limits. Despite what has been said in general terms, some of the sensors have extended response ranges enabling their use in non-habitual conditions. For example, the DS18B20 temperature sensor has a precision of ±0.5 °C in the range from −10 °C to +85 °C; the humidity sensor DHT22 can read %RH from 0 to 99.9 with a precision of ±2% and the BPM280 barometric pressure sensor can sense from 300 to 1050 hPa with an absolute precision of ±1 hPa. Moreover, the chemical and light sensors chosen, have a reliable response if they work at a temperature within the range from −10 to +85 °C and relative humidity from 10 to 95%, except the CO sensor MQ-7 that have an operation temperature somewhat limited, from −20 °C to +50 °C, but enough for the purpose of this work. Manufacturers of such sensors suggest their use in smartphones, GPS modules, watches, wearables, smart homes, indoor and outdoor navigators or weather forecast but they explicitly warn that the sensors should not be used in safety or emergency stop devices or any other occasion that failure of sensor may cause personal injury or material losses.

Furthermore, we propose Bluetooth and Wi-Fi as the technologies that enable wireless exchange of data with external devices (mobile phone, personal computer, laptop or cloud platforms). On the other hand, the experimental framework establishes different data storage and monitoring options: locally on a μSD (Micro Secure Digital), on a Mobile Application, on a Web Server or on a Cloud Server. The transfer of this information is carried out via Bluetooth or via Wi-Fi using an API that exchanges JSON (JavaScript Object Notation) data between the different devices and the SBA.

As a fourth aspect, the framework enables to incorporate auxiliary modules needed for a specific SBA that do not include certain functionalities (such as Bluetooth or Wi-Fi) and additional electronic components (such as voltage dividers or level shifters) for the proper functioning of the system.

Finally, the framework establishes the process of monitoring, evaluation and analysis of results. The monitoring will consist of storing the information extracted from the sensors in a μSD, visualizing them in a mobile application or a personal computer and storing them in a cloud platform. For the evaluation and analysis of the results, the criteria established are power consumption, reliability, flexibility programming, support availability and electronic utilities.

For this purpose, the different options regarding the programming, support and electronic capabilities of the SBAs will be considered during the development phase. Once the system is functional, it should be working under typical environmental conditions of a home or workplace for at least one week, collecting every minute the information provided by the sensors cyclically.

**Figure 3:**
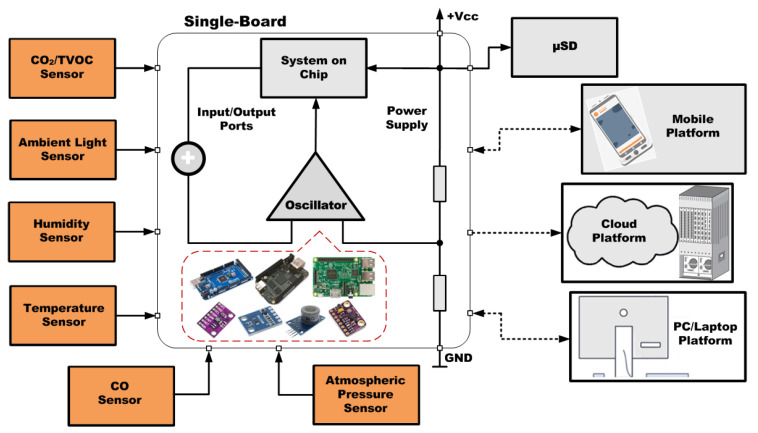
Block Diagram of the Experimental Framework.

Figure 3 shows the scheme of the experimental framework developed, where all the sensor modules are connected to the analyzed single-board, through their respective connection interfaces.

### 3.2. Single-Board Computers and Single-Board Microcontrollers Selection

Currently there is a wide variety of SBAs families on the market, as described in Section 2 above, so we have established a selection criterion, trying to cover the different alternatives and considering aspects as ease of acquisition, user-friendliness, cost, architectures, formats, configurations, families, versions and providers.

**Figure 4:**
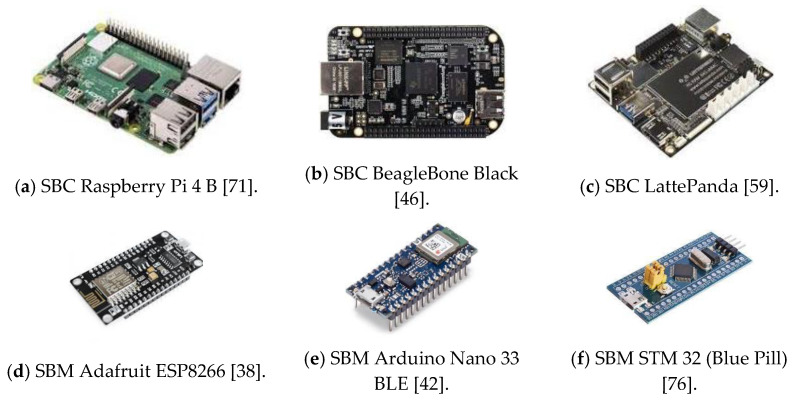
Low-cost Single Boards analyzed in experimental framework.

In this sense, the analysis is carried out with six low-cost single boards: three Single-Board Microcontrollers (SBMs) and three Single-Board Computers (SBCs), they are shown in Figure 4.

The six selected low-cost SBAs are very current versions with cost less than 100€. They will allow us to analyze the sensors on the experimental framework and determine their virtues. Moreover, in a way, from our point of view, this selection of boards provides a standardized comparison scenery of the capabilities and possibilities of the single-board market.

In this sense Raspberry Pi 4 B is the latest version of one of the most popular SBC families, it is very easy to acquire, as it is supplied by a wide variety of distributors and electronics manufacturers. Raspberry Pi is used in IoT and sensor applications and a wide range of projects developed by technology companies, universities and hobbyists/maker community. It is a user-friendly platform with a lot of resources available. It has an ARM (Advanced RISC Machine) architecture with a Linux based OS, which allows the use of different programming languages and IDEs.

Among the variety of single-board computers within the BeagleBoard family, one of the most popular is the BeagleBone Black (BBB), although it is similar to the Raspberry Pi and even they can often be interchangeably, however there are some important electronics characteristics that place them as the best solution in many applications. The most remarkable features of the BBB are its wide set of inputs/outputs (69 GPIO pins), including SPI and I^2^C bus, serial ports, PWM (Pulse Width Modulated) outputs and analog inputs, which facilitate the direct connection of a wide number of sensors.

In the same way, we have selected the SBC LattePanda 2G/32G, within the SBAs that can support a version of Windows OS, since it is one of the cheapest of them. The possibility to implement projects integrating sensor technologies using Windows OS increases the interest of this SBC for a wide variety of technology companies, universities and developers. In addition, this SBC incorporates an Arduino Leonardo internally, which also facilitates implementations based on this platform due to the wide use of Arduino.

In the selection of the three SBMs we have taken into account the size as the main criterion, in contrast with the SBCs chosen. In this case, all the SBMs chosen are nano-sized type. In this selection we have also tried to cover all the possibilities of the SBM market, choosing boards of main manufacturer with different functionalities. Therefore, we have selected Arduino Nano 33 BLE, Adafruit Feather HUZZAH ESP8266 and STM32F103C8T6 (Blue Pill).

The Arduino family is undoubtedly one of the best known and currently used platforms in applications of single board architectures integrating sensors, thus we have chosen Arduino Nano 33 BLE, since it is a small size board with Bluetooth Low Energy (BLE).

Adafruit Feather HUZZAH ESP8266 is a low-cost and small type microcontroller board manufactured by Adafruit Industries [38] integrating a low-cost ESP8266 microcontroller of Espressif Systems Semiconductor Company [52] with Wi-Fi capacity.

The STM32F103C8T6 single-board microcontroller is one of the cheaper versions of the STM family, with excellent electronic features. Even though it does not include Bluetooth or Wi-Fi capabilities, its number of GPIO pins, SPI and I^2^C buses, serial ports and analog inputs make it a suitable option for applications of single board architectures integrating sensors.

**Figure 5:**
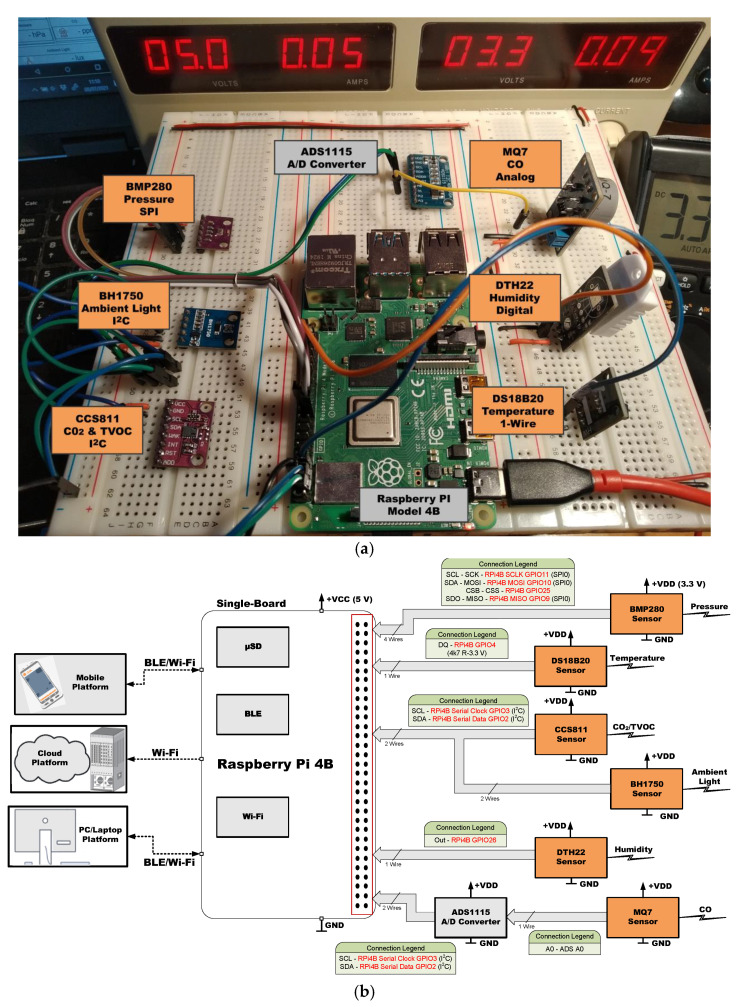
SBC Raspberry Pi 4 B in experimental framework (**a**) and hardware connections (**b**) [150].

**Figure 6:**
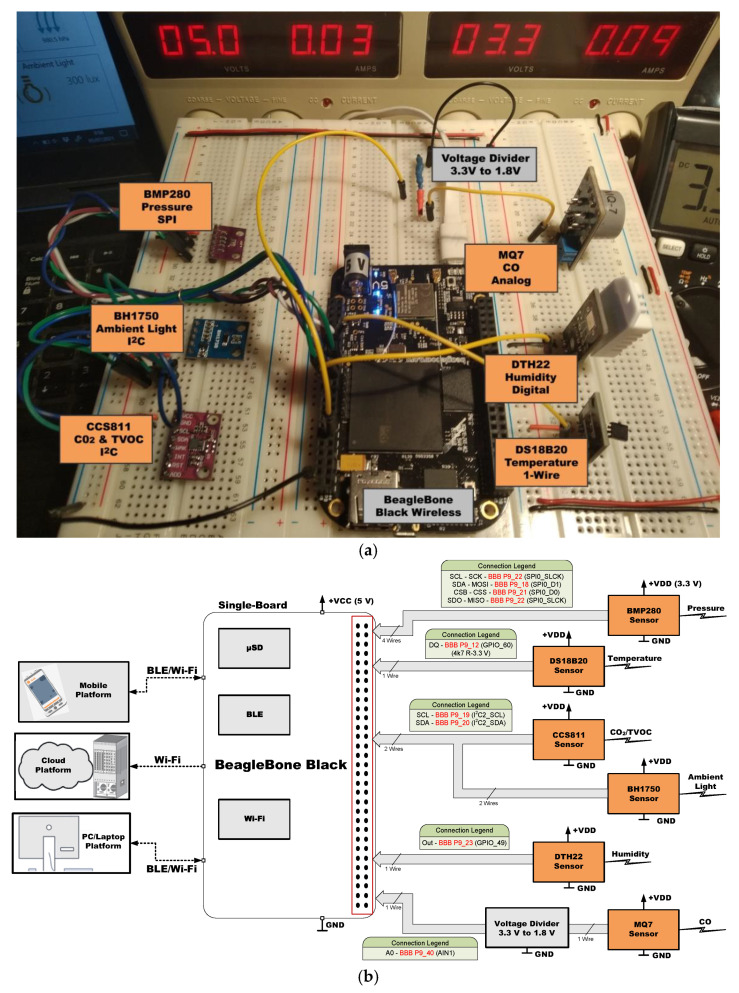
SBC BeagleBone Black in experimental framework (**a**) and hardware connections (**b**) [150].

**Figure 7:**
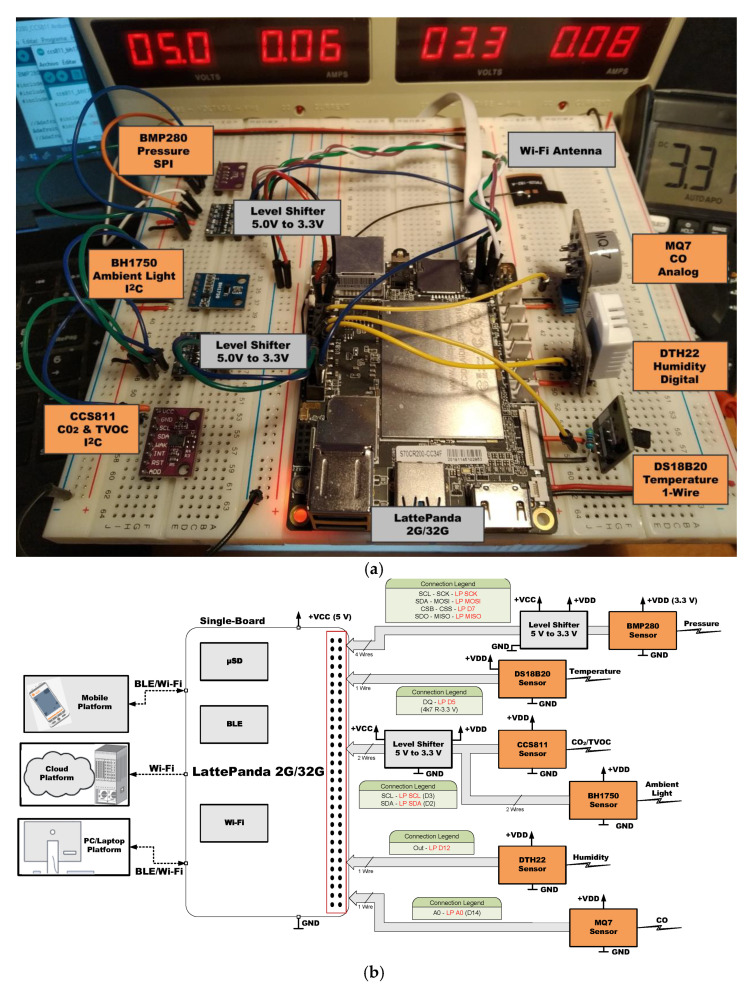
SBC LattePanda 2G/32G in experimental framework (**a**) and hardware connections (**b**) [150].

**Figure 8:**
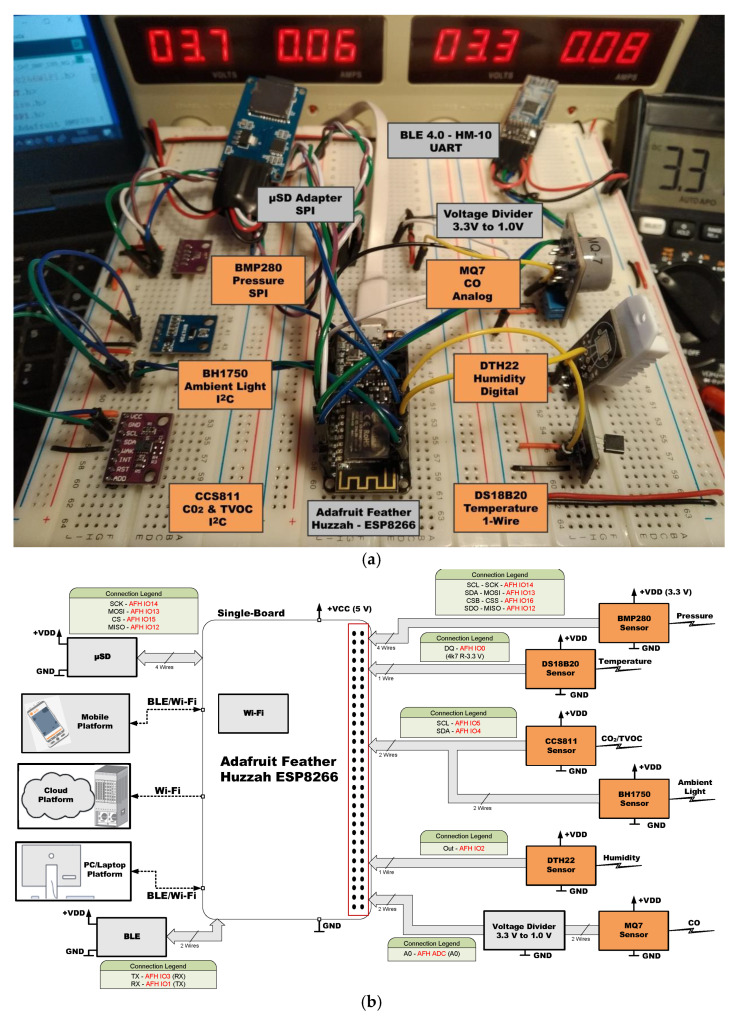
SBM Adafruit Feather Huzzah ESP8266 in experimental framework (**a**) and hardware connections (**b**) [150].

**Figure 9:**
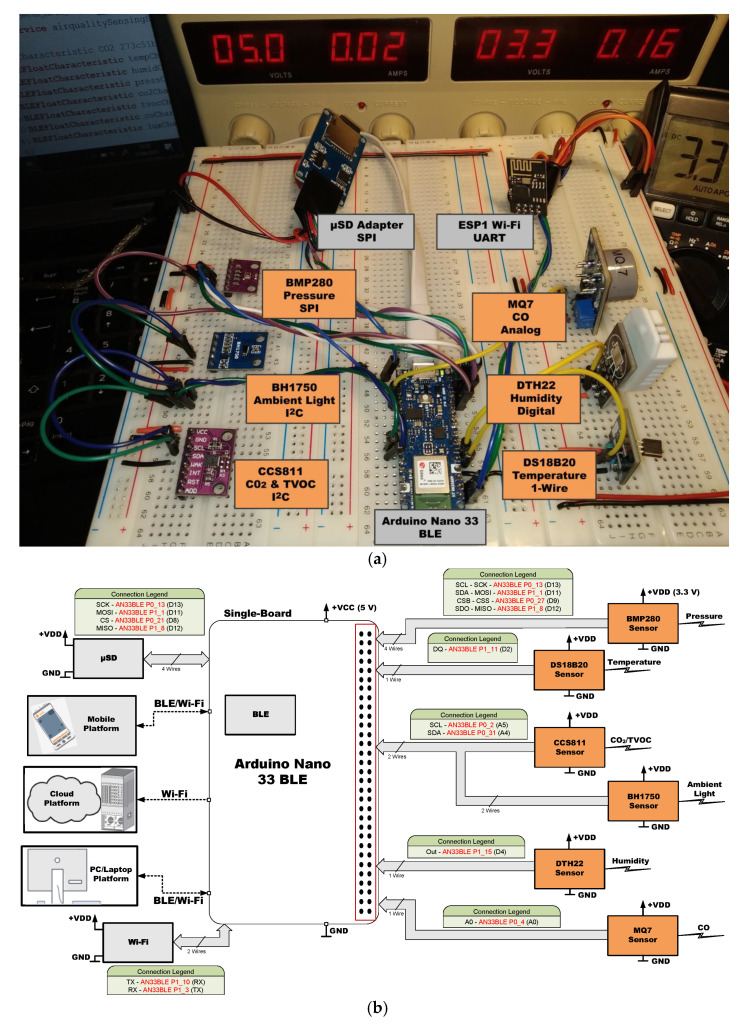
SBM Arduino Nano 33 BLE in experimental framework (**a**) and hardware connections (**b**) [150].

**Figure 10:**
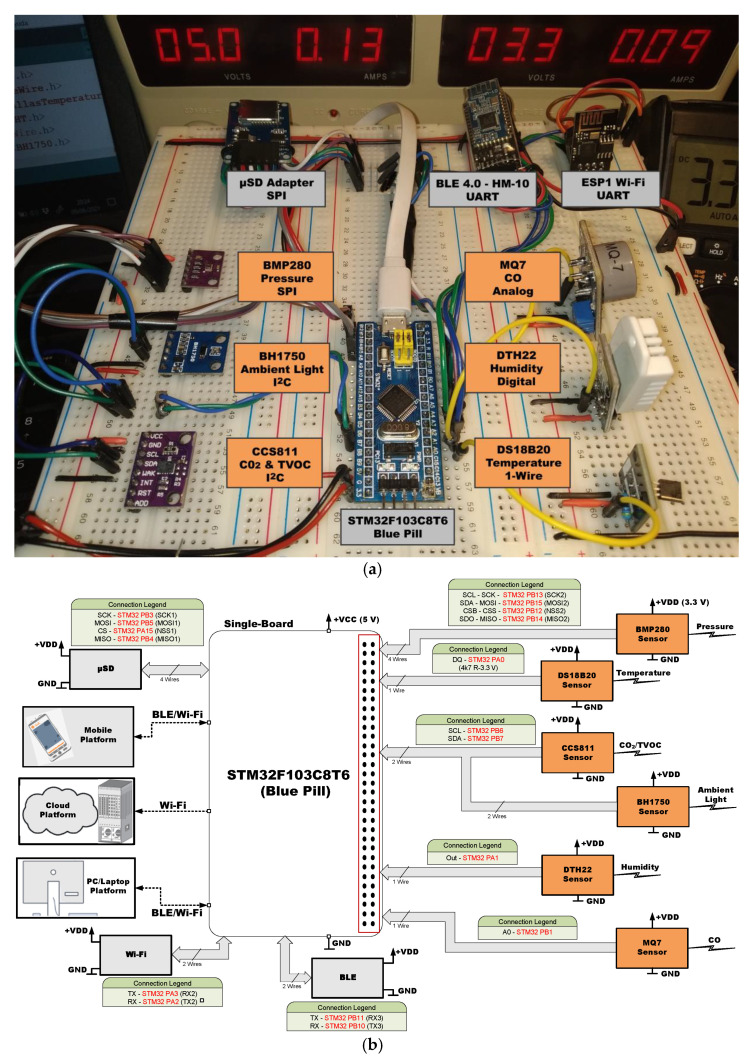
SBM STM32F103C8T6 (Blue Pill) in experimental framework (**a**) and hardware connections (**b**) [150].

Figure 5, Figure 6, Figure 7, Figure 8, Figure 9 and Figure 10 present the analyzed low-cost Single Boards in the experimental framework with the six sensor modules and all the auxiliary devices needed to analyze them as well as the connection diagrams made to connect these SBAs with the set of sensors. More information can be found in the paper’s GitHub repository [150].

### 3.3. SBMs and SBCs Programming

As regards programming languages and development environments, it is necessary to distinguish between SBCs and SBMs. SBCs can be programmed directly on the single-board itself since they have an operating system. These developments can be carried out with a wide variety of programming languages (C, C++, Java, Python, JavaScript and other more) and with different IDEs. On the other hand, SBMs are more limited in this sense; generally, C programming language (or some derivative) is used, and IDEs such as Arduino IDE [151] for a wide variety of SBMs, Mbed Compiler [152] for single-boards with ARM microprocessors, or STM32 IDEs [153,154] for STM32 family.

In the experimental framework and once analyzed the advantages and disadvantages of each of the available programming languages and IDEs, we have used Python as programming language due to its advantages such as libraries, support, functionality and we have used Visual Studio Code [155] for the three SBCs analyzed; while for the SBMs we have used Arduino IDE and C programming language, mainly due to Arduino community’s support. All developed source codes are available in a publish-only GitHub repository [150].

In order to test the designed experimental framework, we have developed software applications such as a Mobile App, a Web Interface and we have used a Cloud Service that allow the connection with SBAs via Bluetooth and Wi-Fi, for the transfer of the data obtained by the sensors.

**Figure 11:**
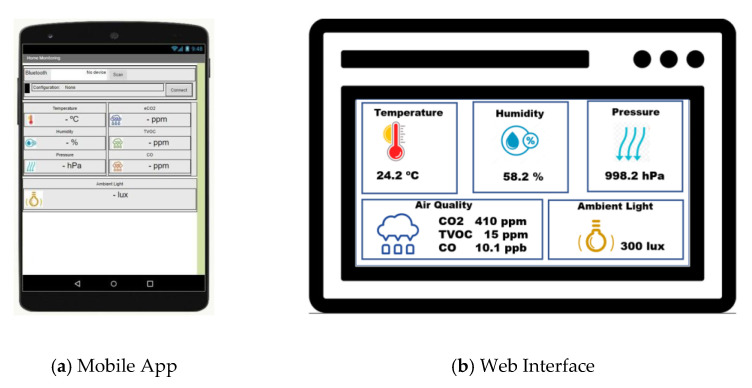
Mobile App and Web Interface developed for the experimental framework.

The Mobile App has been developed with MIT App Inventor [156] of the Massachusetts Institute of Technology. The graphical interface of this application is shown in Figure 11a. This App has been designed for monitoring the provided data by the SBAs via Bluetooth or via Web.

The Web Interface has been designed with standard web technologies: HTML (Hyper Text Markup Language), CSS (Cascading Style Sheets) and JavaScript. The interface has been developed for monitoring the provided data by the SBAs via an API Web as shown Figure 11b. 

**Figure 12:**
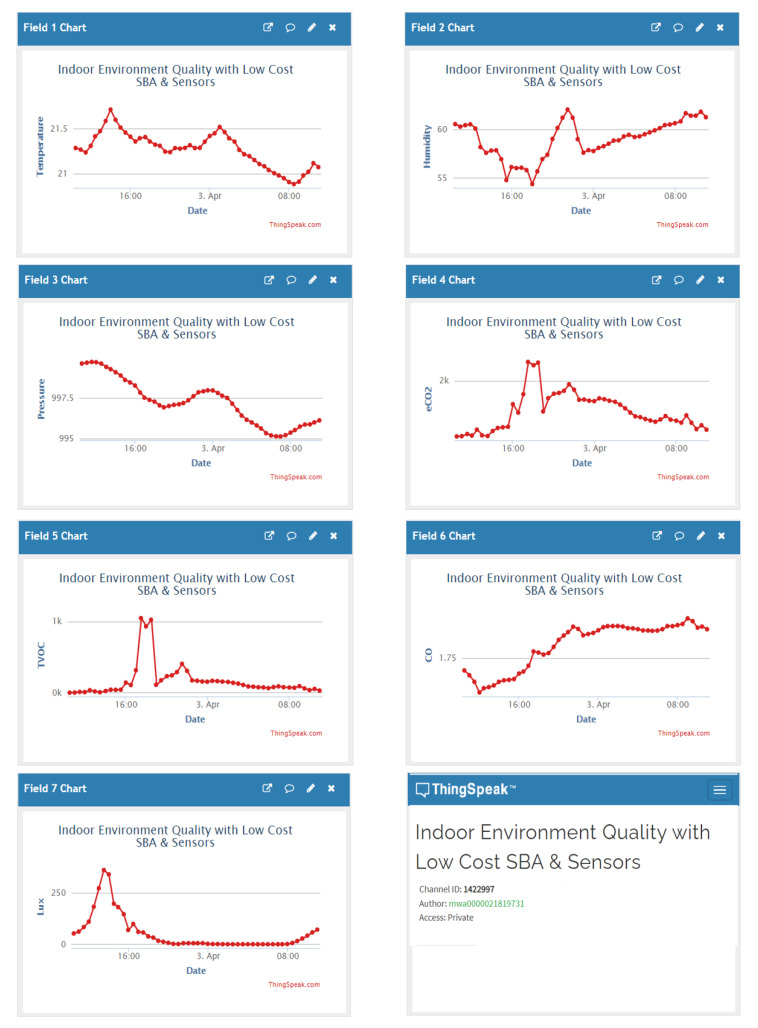
ThingSpeak channel catching the sensors data.

Finally, we have used ThingSpeak [157] as Cloud Service. This open-source service is used on Internet of Things to store and collect data from objects connected through the Internet and the Hypertext Transfer Protocol (HTTP). With ThingSpeak, a record of the data provided by the sensors has been created, which allows the analysis, observation and evolution over time of the measured variables by mean a graphic representation. (Figure 12).

### 3.4. Experimental Results

In this Subsection we show the results obtained in the experimental framework described in Section 3.1 with the six SBAs presented in Section 3.2 and we introduce the most important characteristics to evaluate.

From a general viewpoint, there are some characteristics that should be analyzed when choosing the most suitable SBA for a particular application. The most important characteristics that we have considered in the experimental results are Power Consumption (mA@V), Reliability (%), Programming Flexibility, Support Availability and Electronics Utilities.

Since SBA based systems integrating sensors are usually powered by batteries and should operate for long periods, power consumption is a relevant characteristic which must be evaluated. Therefore, low power consumption systems are preferable.

On the other hand, in this experimental framework, we consider reliability as the system’s tolerance to failures in the processing of the information provided by the sensors, specifically calculated as the complementary (1 − ε) of the error number (ε) in sensor reading or data transmission. When several sensors are connected the system must maintain reliability, so obviously, systems with fewer errors are the most suitable.

In order to evaluate the programming flexibility of SBAs, we have considered the availability of programming languages and libraries as well as development environments and other utilities. This characteristic was assessed by means of a qualitative variable with three grades: high, medium and low.

We are considered the support availability as the possibility of programming SBAs with low prior knowledge, easy to learn and easy to handle, as well as the relevance of the developer community around the platform and the links with developed projects. This characteristic is also evaluated by means of a qualitative variable with three grades: high, medium and low.

Each SBA has its own architecture including a specific number of GPIO, analog and PWM pins, SPI and I^2^C buses, serial communication ports, output voltage pins and other more. Thus, we consider electronics utilities characteristic as a measure of the electronic capability of each SBA, evaluated as a qualitative variable with five grades: highest, high, medium, low and lowest.

The experimental results have been obtained after a previous development and evaluation analysis. All the SBAs have been kept running for one week in the experimental framework. As a conclusion, a summary of the results obtained is shown in Table 10, where the main characteristics are presented: Power Consumption, Reliability, Programming Flexibility, Support Availability and Electronics Utilities.

Reliability has been measured by analyzing the number of errors during the operating time. Programming flexibility has been considered from experience during development time. Support availability has been obtained by taking into account the web positioning on the Internet, of the families of SBAs established as the relevance of the community and the number of developed projects. Electronics utilities characteristic has been determined considering the different functionalities of each SBA. Finally, power consumption average has been measured at supplied current conditions in relation to the supply voltage with the six sensors, Wi-Fi and BLE devices connected to each of the SBAs, according to a commercial power supply monitor (with 3.3 V and 5 V). We highlight that a current peak occurs, which can be as high as 300 mA, at the start-up time of the Wi-Fi module.

### 3.5. Discusion

All SBAs and the sensors were analyzed in experimental framework by means developed prototypes. In this subsection, the results will be discussed. In this sense all SBCs have integrated μSD, Wi-Fi and BLE modules. On the other hand, the SBMs have been expanded with a standard low-cost μSD card adapter connected with SPI protocol. Moreover, the SBMs that do not have Wi-Fi and BLE functionalities have been extended with ESP1 and HM-10 modules, respectively. ESP1 module contains a low-cost ESP8266 microcontroller with integrated Wi-Fi manufactured by Espressif [158]. HM-10 module includes a low energy SoC based on CC2541 for BLE applications [159]. In the same way, Raspberry Pi 4 does not have a way to read analog inputs. This causes the connection of some sensors to require the use of an external ADC. Even though there are different solutions, we have opted to use the ADS1115 device manufactured by Texas Instruments [160], it is a low-power, I^2^C-compatible, 4-channel, 16-bits resolution converter. In addition, the analog input pins of BBB and AF ESP8266 single-boards can only accept up to 1.8 V and 1.0 V maximum voltage, respectively, this involves that the voltages produced by the analog sensors must be adapted.

Taking into account the results obtained in the experimental framework and shown in Table 10, our analysis concludes that the three most robust SBAs are BBB, AN 33 BLE and Blue Pill since the error obtained in our analysis is less than 0.01%. LP 2G/32G, RPi 4 and Blue Pill single-boards present the most programming flexibility since they have a wide variety of programming languages and libraries available as well as development environments and other utilities. RPi 4 and AN 33 BLE provide the best support availability since they allow the development of projects with low prior knowledge, easy to learn and easy to handle, as well as the great relevance of the developer community around these platforms. Additionally, both BBB and Blue Pill have the best electronic utilities due to their greater capabilities and functionalities in project development. Finally, regarding the measured power consumption average, and independently of the number of connected sensors, the SBAs show a higher consumption, but they have also a higher number of integrated resources (μSD, Wi-Fi and BLE); in this sense obviously, the three SBCs show a higher consumption than the three SBMs analyzed, with the LP 2G/32G showing the highest consumption.

Table 11 summarizes the expansion modules and devices that must be used to complete the framework enabling that the same group of sensors can be implemented with the SBAs selected in our analysis. As can be seen, each SBA needs its own solution, but all the modules and devices used can be acquired by a low-cost and are fully compatible with all sceneries.

## 4. Conclusions

In this paper we realized an analysis of the most important topics related with single-board architectures integrating low-cost sensors. Our goal is to highlight some of the main features and possibilities that these architectures present today, as well as their evaluation and comparison. We analyze the most popular platforms based on characteristics as: cost, processing capacity, integrated processing technology and open-source license, as well as power consumption, reliability, programming flexibility, support availability and electronics utilities. For evaluation, an experimental framework has been designed and implemented with six sensors (temperature, humidity, CO_2_/TVOC, pressure, ambient light and CO) and different data storage and monitoring options: locally on a μSD, on a Cloud Server, on a Web Server or on a Mobile Application. The experimental framework allows discussing the most important aspects that involves the design, development and implementation of a project by means low-cost single board architectures integrating low-cost sensors based on the obtained results.

## Figures and Tables

**Table 1:** Main proprietary SBMs in the market.

	ST Microcontroller (MCU) [76]		Texas Instruments [78]	
Processor	STM32	STM8	MSP430	AM65x/AM572x/DRA821xM
Architecture / Bits	Arm Cortex-M/32	Harvard MCU/8	RISC/16	Arm Cortex-M4F/32
Europe Prize (€) [44,49,54,72]	<20	~40	10–20	2.5–10
IDE	STM32Cube	STVD-STM8	Energia	CCStudio
Open-Source HW	No	No	No	No
Open-Source SW	Yes	Yes	Yes	No
High Perform. Versions	386	-	-	-
Mainstream Versions	353	41	-	8
Low Power Versions	341	22	10	-
IST	Yes	Yes	Yes	Yes
Most Popular	STM32F103C8T6	STM8L15X	TMS320C6457	
	STM32F107V/C			
	STM32F303VCT6			
	STM32F439ZI			
	STM32F756ZI	STM8L152C6		
	STM32L010RB			
	STM32L4A6ZG			
	STM32L4R5ZI/-P			

**Table 2:** Main open-source SBMs in the market.

	Wiring [85]	Adafruit [38]	Arduino/Genuino [42]		Teensy [77]
Processor	AVR8	Tensilica L106	AVR8	ARM Cortex-M0+	ARM Cortex-M
Architecture/Bits	AVR atmega/8	RISC/32	AVR atmega/8/32	Atmel SAMD21/32	MK20DX/32
Europe Prize (€) [44,49,54,72]	-	10	10–35	20–40	10–30
IDE	Wiring	Arduino IDE MicroPyton	Arduino IDE	Arduino IDE	Teensyduino
Open-Source HW	Yes	Yes	Yes	Yes	Yes
Open-Source SW	Yes	Yes	Yes	Yes	Yes
Versions	3	1	10	11	8
IST	No	No	Yes	Yes	No
Most Popular		Huzzah ESP8266	UNO Rev. 3		Teensy LC
	Wiring V1.1		Mega 2560	MKR1000	Teensy 3.2
	Wiring Mini V1.0		Leonardo	MKR Zero	Teensy 3.6
	Wiring S		Nano Every	Zero	Teensy 4.0
			Micro		Teensy 4.1

**Table 3:** The SBC Raspberry PI Model B family [71].

Model	Zero W	1 B+	2 B	3 B+	4 B
SoC	BCM2835	BCM2835	BCM2836	BCM2837B0	BCM2711
Processor/Cores/Bits	ARM11/1/32	ARM11/1/32	Cortex A7/4/32	Cortex A53/4/64	Cortex A72/4/64
Frequency (GHz)	1.0	0.7	0.9	1.4	1.5
RAM (GB)	0.5	0.5	1.0	1.0	2/8
Wireless	Wi-Fi, BT, BLE	No	No	Wi-Fi, BT, BLE	Wi-Fi, BT, BLE
Connectivity	HMDI, USB, µUSB, Video RGB, CSI Cam	HMDI, USB 2.0, Ethernet, Audio, Video RGB	HDMI, USB 2.0, Ethernet, Audio, Video RGB, CSI Cam	HMDI, USB 2.0, µUSB, Ethernet, Audio, Video RGB, CSI Cam	µHMDI, USB 3.0, USB-C, Ethernet, Audio, Video RGB, CSI Cam
OS	NOOBS and Linux				
Europe Prize (€) [44,49,54,72]	15	32	44	43	40/84

**Table 4:** Main open-source SBCs Linux based in the market.

Model	BeagleBone Black [46]	Odroid XU4 [66]	Tinker Board 2 [79]
SoC	Sitara AM3358	Exynos5422	Rockchip RK3288
Processor/Cores/Bits	ARM Cortex-A8/1/32	ARM Cortex-A15/4/32	ARM Cortex-A17/4/64
Frequency (GHz)	1.0	2.0	1.8
RAM (GB)	0.5	2.0	2.0
Wireless	-	Wi-Fi (option)	Wi-Fi, BT
Connectivity	USB 2.0, Ethernet, UART, SPI, I^2^C, HDMI, CAN	USB 2.0/3.0, Ethernet, UART, SPI, I^2^C, HDMI	USB 2.0, Ethernet, UART, SPI, I^2^C HMDI, SD 3.0
OS	Debian and ROS	Ubuntu 16.04 and Android 4.4.x	Debian and Android
Europe Prize (€) [44,49,54,72]	65	60	70

**Table 5:** Main open-source SBCs Windows based in the market.

Model	LattePanda 2G/32Gb [59]	DragonBoard410c [44]	Udoo x86 Ultra [82]
SoC	Intel HD Gen8	Qualcomm APQ8016E	Intel HD Graphics 405
Processor/Cores/Bits	Intel Atom X5/4/64	ARM Cortex-A53/4/32/64	Intel Pentium x86/4/64
Frequency (GHz)	1.8	1.2	2.56
RAM (GB)	2.0	1.0	8.0
Wireless	Wi-Fi, BT	Wi-Fi, BT	Wi-Fi slot
Connectivity	USB 2.0 / 3.0, Arduino GPIO, Ethernet, UART, I^2^C, HDMI, µSD, CAN, Audio	USB 2.0, Ethernet, UART, SPI, I^2^C, HMDI, GPS	USB 3.0, Arduino GPIO, Ethernet, UART, HDMI, µSD, Audio
OS	Windows 10	Android, Linux and Windows IoT	Android, Linux and Windows 10
Europe Prize (€) [44,49,54,72]	60	60	250

**Table 6:** Open-source SBAs with FPGA.

Model	Papilio DUO [135]	Alchitry Au [136]	Alhambra II [137]	MKR Vidor 4000 [138]
Processor	ATmega32U4	-	-	Cortex-M0 SAMD21
FPGA type	Spartan 6	Artix 7	iCE40	Cyclone 10CL016
Flash (MB)	64	-	32	2
RAM (MB)	2	256	-	8
GPIO (pins)	54	102	12	22
IDE (processor)	Arduino	-	-	Arduino
IDE (FPGA)	EDK, Chipscope, Impact	EDK, Chipscope, Impact	iCEstudio	Quartus
Europe Prize (€) [44,49,54,72]	300	85	50	63

**Table 8:** Low-cost sensors used in experimental framework.

Sensor	Measured Variable	Connection Type	Reference Manufacturer
DS18B20	Temperature	1-Wire	Maxim/Dallas [144]
DHT22	Humidity	Digital Output	Aosong [145]
CCS811	CO_2_/TVOC	I^2^C Interface	Ams AG [146]
BMP280	Pressure	SPI	Bosch Sensortec [147]
BH1750	Ambient Light	I^2^C Interface	Rohm [148]
MQ-7	CO	Analog Output	Hanwei [149]

**Table 9:** Power Consumption and Cost of sensors in experimental framework.

Sensor	Supply Voltage (V)	Supply Current (mA)	Module Cost (€)
DS18B20	+3.0 to +5.5	<1 mA@3.3V	≈2.00€
DHT22	+3.3 to +5.5	<1 mA@3.3V	≈3.00€
CCS811	+1.8 to +3.6	<1 mA@3.3V	≈5.00€
BMP280	+1.7 to +3.6	<1 mA@3.3V	≈1.00€
BH1750	+2.4 to +3.6	<1 mA@3.3V	≈1.00€
MQ-7	+2.5 to +5.0	80 mA@3.3V	≈1.50€

**Table 10:** Results obtained in the experimental framework with SBAs: Raspberry Pi 4 B (RPi 4), BeagleBone Black (BBB), LattePanda 2G/32G (LP 2G/32G), Arduino Nano 33 BLE (AN 33 BLE), Adafruit Feather HUZZAH ESP8266 (AF ESP8266) and STM32F103C8T6 (Blue Pill).

SBA	Reliability	Programming Flexibility	Support Availability	Electronics Utilities	Power Consumption
RPi 4	98%	High	High	High	≈2.5 W
BBB	99%	Medium	Medium	Highest	≈1.7 W
LP 2G/32G	95%	High	Low	Medium	≈5.0 W
AN 33 BLE	99%	Medium	High	High	≈700 mW
AF ESP8266	98%	Medium	Medium	Medium	≈630 mW
Blue Pill	99%	High	Medium	Highest	≈730 mW

**Table 11:** Modules used with each SBAs in experimental framework.

SBA	µSD	Wi-Fi	BLE	ADC	Level Shifter	Voltage Divider
RPi 4				✓		
BBB						✓
LP 2G/32G					✓	
AN 33 BLE	✓	✓				
AF ESP8266	✓		✓			✓
Blue Pill	✓	✓	✓			

## Data Availability

Not applicable.
